# Examining the Association Between Electrodermal Activity and Problem Behavior in Severe Autism Spectrum Disorder: A Feasibility Study

**DOI:** 10.3389/fpsyt.2019.00654

**Published:** 2019-09-11

**Authors:** Bradley J. Ferguson, Theresa Hamlin, Johanna F. Lantz, Tania Villavicencio, John Coles, David Q. Beversdorf

**Affiliations:** ^1^Department of Health Psychology, University of Missouri School of Health Professions, Columbia, MO, United States; ^2^Thompson Center for Autism & Neurodevelopmental Disorders, University of Missouri, Columbia, MO, United States; ^3^Department of Radiology, University of Missouri School of Medicine, Columbia, MO, United States; ^4^The Center for Discovery, Harris, NY, United States; ^5^CUBRC, Inc. Information Exploitation Sector, Buffalo, NY, United States; ^6^Department of Industrial and Systems Engineering, University at Buffalo, Buffalo, NY, United States; ^7^University of Missouri Departments of Neurology & Psychological Sciences, Columbia, MO, United States

**Keywords:** autism spectrum disorder (ASD), electrodermal activity (EDA), skin conductance, problem behavior, stress, anxiety, intellectual disability

## Abstract

**Background:** Many individuals with autism spectrum disorder (ASD) engage in problem behavior, presenting significant challenges for those providing care and services for this population. Psychophysiological measures of arousal, such as electrodermal activity (EDA), may provide an early indication of subsequent problem behavior. However, variability in EDA patterns associated with behaviors may limit this predictive ability.

**Methods:** EDA data was sampled from eight individuals with severe ASD in a naturalistic setting, while participating in educational programming in a school setting at a residential facility for severely affected individuals with developmental disabilities, to examine variability in EDA patterns.

**Results:** An anticipatory rise in EDA only occurred 60% of the time prior to the problem behavior. Additionally, EDA after a problem behavior returned to median baseline levels only 45% of the time.

**Conclusions:** Heterogeneity of EDA responses in those with the most severe forms of ASD will be an important consideration in future studies utilizing psychophysiological tools such as EDA to anticipate problem behavior, including the need for monitoring of return to baseline after problem behaviors. Incorporation of this consideration may lead to greater reliability of these approaches to help anticipate and manage problem behaviors.

## Introduction

Autism spectrum disorder (ASD) is a neurodevelopmental condition that is characterized by persistent deficits in social communication and restricted, repetitive patterns of behavior that occur early in development (1). Research has demonstrated that many individuals with ASD engage in aggression and other problem behaviors such as self-injurious behavior and irritability. For example, one study found that in a sample of 1,380 children with ASD, up to two-thirds became aggressive toward their caregivers, and nearly half became aggressive toward others (2). Those individuals with ASD with greater intellectual impairment, often with significantly limited communication and social engagement skills, are at greater risk for the development of problem behaviors (3). Problem behavior poses many challenges for caregivers and service providers alike, with the potential to become worse over time (4). In order to attempt to reduce the occurrence of problem behavior, research has begun to explore the use of psychophysiological markers preceding problem behavior so that an intervention may occur prior to the onset of the behavior.

Among those with ASD, high levels of stress may manifest as problem behaviour (5). Additionally, emotional regulation appears to be impacted in general in ASD (6). Knowing the physiological stress state of individuals with ASD may allow caretakers and service providers to intervene prior to the occurrence of problem behavior. Furthermore, better understanding environmental correlates of physiological responses can help to develop more precise treatments through control of or manipulation of these factors. One psychophysiological technique to assess an individuals’ internal stress state is the measurement of electrodermal activity (EDA). Increases in EDA indicate activation of the sympathetic nervous system (the “fight-or-flight” response of the autonomic nervous system), which is measured by assessing changes in electrical conductivity between two electrodes placed in close proximity to each other on the skin. Activation of the sympathetic nervous system results in secretion of sweat, which conducts electricity, from eccrine sweat glands throughout the body. Eccrine sweat glands are only innervated by the sympathetic branch of the autonomic nervous system, so increases in EDA can be, in part, attributed to increases in physiological arousal. EDA is typically measured from areas of the body with a high density of eccrine sweat glands, such as the palms of the hands or soles of the feet, but can also reliably measure sweat secretion when placed on other areas of the body such as the wrist or immediately above ankle (7). Measurement of EDA has been shown to be well-tolerated in ASD and is sensitive to changes in arousal and emotional states in this population (8–10). However, changes in EDA in response to an arousing stimulus, vary widely in ASD. For example, abnormal baseline EDA as well as both hypo- and hyperactivities in response to human faces have been shown in ASD (11). Typically, EDA returns to baseline shortly after the application of an arousing stimulus. However, some children with ASD fail to return to their baseline EDA after the occurrence of an environmental stimulus (11), suggesting that a large stress response may continue to affect behavior long after the occurrence of a stressful event, including the engagement in problem behavior. A recent study found a relationship between EDA and externalizing behavior problems during compliance-oriented play tasks (12). However, EDA was relatively low while the individual engaged in the problem behavior, suggesting heterogeneity in the autonomic nervous system response to task demands in ASD. Furthermore, greater variability in EDA in response to a battery of naturalistic and structured parent–child, child alone, and direct testing tasks has been shown to be associated with overall ASD severity (13), suggesting that EDA may be a promising predictor of problem behavior in ASD. In light of this, research is now beginning to explore the utility of psychophysiological markers to anticipate problem behaviors in ASD. One recent study has utilized multimodal psychophysiological arousal to successfully predict aggression in minimally verbal ASD patients in a naturalistic setting (14).

A great amount of heterogeneity exists in ASD, which can complicate research efforts when examining data at the group level. As such, identification of subtypes within ASD may lead to more effective treatments (15, 16). Psychophysiological data has recently been used to identify relationships between co-occurring symptoms in ASD, such as gastrointestinal symptoms, irritability, and sleep problems (8). Therefore, while psychophysiological data may also be useful to assess the internal stress state of individuals with ASD in a variety of settings, it is important to examine the heterogeneity of this response. Understanding this variability will be important in the development of tools to anticipate the onset of behaviors, which may help lead to more individualized treatment approaches. This will be of a particularly acute need in the most severely affected individuals, for whom problem behaviors are more frequent (3).

The present study first examined the feasibility of collecting EDA data from individuals with severe ASD in a naturalistic setting, while participating in skill acquisition in a school setting at a residential facility for severely affected individuals with developmental disabilities. The lab school at The Center for Discovery (TCFD) utilizes discreetly mounted video cameras and microphones in classrooms to collect behavioral data, while students wear physiological data collection sensors to gain a better understanding of the physiological correlates associated with learning and behavior in individuals with ASD. As such, we wished to examine the feasibility of examining psychophysiological variables in individuals with severe ASD as they are related to problem behavior. The beginning and end of problem behaviors were identified through video recordings and were confirmed by trained staff at TCFD. The associated EDA (i.e., time locked to the EDA recordings) was monitored prior to the occurrence of the initial problem behaviors as well as immediately after the cessation of the problem behavior, to examine individual variability in this particular psychophysiological variable as it related to problem behavior.

## Methods

### Participants

Eight individuals with ASD (age = 15.9 years ± 2.5 std. dev., range = 13–20, all male, 6 Caucasians, 2 Hispanics) were examined (see **Table 1** for descriptive statistics). Diagnosis of ASD was made by a licensed psychologist and corroborated with scores from the Autism Spectrum Rating Scales (ASRS) DSM-IV-TR scale (17), a 70-item parental report of how often their child displayed each behavior associated with ASD in the past 4 weeks derived from DSM-IV-TR criteria for ASD (17). The ASRS is administered annually for all students. ASRS scores for this study correspond to the year of EDA data collection. The students’ scores from the ASRS DSM-IV-TR scale all fell within the elevated (65–69) or very elevated (70–85) ranges (mean 76.5 ± 6.0 std. dev.) (**Table 1**), supporting that the students all had symptoms directly related to the DSM-IV-TR diagnostic criteria for ASD. Intelligence quotient (IQ) scores were obtained from administration of either the Stanford–Binet Intelligence Scales—5^th^ Edition for students with verbal abilities (18), or the Comprehensive Test of Non-Verbal Intelligence—2^nd^ Edition (CTONI-2) for those who were non-verbal (19). IQ scores were able to be obtained from only five of the eight students due to compliance issues. For those five students, IQ scores were 3.6 standard deviations below the mean (mean IQ 46.6 ± 5.5 std. dev.). Furthermore, adaptive behaviors were assessed by the Adaptive Behavior Assessment System (ABAS) General Adaptive Composite (20) score or the Vineland Adaptive Behavior Scales (VABS) Adaptive Behavior Composite (21) score. As both measures were normalized to a mean of 100, an average score was calculated as an overall estimate of adaptive behavior. On average, the adaptive behavior scores for the students were 3.8 standard deviations below the mean (mean 43.0 ± 7.4 std. dev.).

**Table 1:** Participant demographics and descriptive statistics.

ID	Age	Gender	Ethnicity	Intelligence quotient (type) (*M* = 100, *SD* = 15)	Adaptive functioning	ASRS *T*-score (*M* = 50, *SD* = 10)	Co-occurring conditions
S01	18	M	Caucasian	44 (NV)	41 (ABAS)	82	OCD, ADHD, constipation
S06	15	M	Caucasian	52 (SB abbreviated)	45 (VAB)	84	OCD, ADHD, vomiting
S07	13	M	Caucasian	42 (NV)	35 (VAB)	74	Constipation
S08	15	M	Caucasian	NS	38 (VAB)	85	Movement disorder
S09	13	M	Hispanic	NS	59 (VAB)	68	None
S11	15	M	Caucasian	NS	35 (VAB)	76	Constipation, ADHD, GERD
S12	20	M	Caucasian	53 (NV)	48 (ABAS)	70	Constipation
S13	18	M	Hispanic	42 (NV)	43 (ABAS)	73	Constipation

NV, non-verbal; SB, Stanford–Binet; NS, attempted but unable to obtain score; ABAS, Adaptive Behavior Assessment System General Adaptive Composite; VAB, Vineland Adaptive Behavior Scales Adaptive Behavior Composite; OCD, obsessive-compulsive disorder; ADHD, attention-deficit hyperactivity disorder; ODD, oppositional defiant disorder; ASRS, Autism Spectrum Rating Scales; GERD, gastroesophageal reflux disease.

The study was approved by the Institutional Review Board at TCFD. All data were collected from students with ASD enrolled at the TCFD, located in Hurleyville, NY. Due to the severity of the patient population, consent was provided by the parent/legal guardian, and assent was also obtained for those with the capacity to respond after explanation through an explicit social story. The data were processed and analyzed at the University of Missouri, which was approved by the Health Sciences Institutional Review Board through a reliance agreement with TCFD. Research staff at TCFD recorded problem behavior and EDA data for the eight students over 1 year in the lab school setting at TCFD.

### Behavior Monitoring

Monitoring was done while students were engaged in a classroom-based behavior intervention plan, where interventions were targeting specific behaviors. Occurrences of each problem behavior were noted by educational and residential staff trained to document these behaviors, as part of the standard procedure. The onset and offset of the each problem behavior was notated in Noldus The Observer XT software (Noldus Information Technology, Leesburg, VA). The resulting onset and offset of problem behaviors were then used as endpoints for the EDA analysis.

For the EDA analysis, only the first occurrence of a problem behavior was analyzed per session. This was implemented to prevent the occurrence of additive effects of previous problem behaviors on EDA, which may confound the results. Thus, if a participant engaged in problem behavior at 10 min into a session, then EDA analyses were only carried out on that problem behavior, and subsequent problem behaviors during the session were not analyzed. Overall behavioral profile for each student was also tracked, as documented by staff, for all behaviors at any point during the session (see **Table 2** for distribution of behaviors among students).

**Table 2:** Frequency and percentage of the occurrence of an anticipatory rise in EDA prior to the problem behavior, mean anticipatory rise time, number of times that a student returned to baseline EDA after engaging in problem behavior, and the mean time for EDA to return to baseline. Note for S13, no statistics for anticipatory rise and return to baseline were able to be calculated as the student’s repetitive behavior was continuous for the duration of each session.

ID	PB assessed	Valid EDA records	Number of times anticipatory rise prior to PB (%)	Mean EDA prior to PB (µS) (SD)	Mean anticipatory rise time (s) (SD)	Number of times returned to BL after PB (%)	Mean time to return to BL (s) (SD)
S1	Jumping in seat	9	6 (67%)	0.99 (0.85)	590 (466)	5 (56%)	2,165(673)
S6	Repetitive body hitting	11	6 (55%)	1.09 (1.66)	1,076 (1099)	6 (55%)	2,436 (1, 919)
S7	General classroom disruption	9	8 (89%)	2.22 (1.92)	945 (1, 201)	2 (22%)	4,939 (2, 742)
S8	Aggression	8	1 (13%)	0.74 (0.81)	681 (0)	2 (25%)	3,759 (356)
S9	Out of seat	9	5 (56%)	0.48 (0.42)	403 (399)	8 (89%)	6,389 (4, 289)
S11	Self-injurious behavior	9	8 (89%)	0.89 (0.61)	490 (385)	5 (56%)	3,536 (4, 138)
S12	Agitation	7	3 (43%)	0.18 (0.20)	95 (86)	0 (0%)	X
S13	Repetitive motor movement	X	X	X	X	X	X
TOTAL	8	62	37 (60%)	0.94 (0.64)	611 (306)	28 (45%)	3,870 (1, 586)

s, seconds; SD, standard deviation; BL, median baseline EDA level; PB, problem behavior; µS, microsiemens.

### Electrodermal Activity Procedures

EDA data were obtained using a Q-Sensor pod wristband (Affectiva Inc., Cambridge, MA), with a sampling rate of 32Hz, which was placed with Ag/AgCl dry electrodes, for extended use, to the wrist of the non-dominant hand. If the student would not tolerate a wrist-worn sensor, then the sensor was applied to the student’s ankle (22). Once the EDA site was selected (i.e., wrist or ankle), the sensor was always applied to the same site for each child and across time. Though the ankle is not the most optimal location from which to collect EDA (23), this location has been used in previous research in individuals with neurodevelopmental disorders when tolerance to a biosensor is an issue (24). All Q-sensor data were obtained while the students were in a temperature-controlled classroom with the classroom thermostat kept at a consistent temperature for the duration of the study. During baseline data collection, all students were expected to stationary and in their seats performing classroom activities. The Q-sensor devices were time-synchronized daily with the same computer system that has the video recording software to ensure synchronization. At the start of each video session, the Q-sensor button was pressed at the time of recording as an additional time-stamp. Next, EDA data from the Q-Sensor were downloaded onto a computer and processed using Affectiva Software (Affectiva Inc., Cambridge, MA). The EDA data were visually inspected by the first author, who has over a decade of experience working with EDA data, for significant motion artifacts within 5 min of the start of the problem behavior, during the problem behavior, and immediately after each problem behavior. Visual inspection of the EDA data was critical for this particular study due to the severity of the patient population resulting in frequent issues with behaviors resulting in the potential for significant artifacts due to motion. EDA data were excluded from the final analysis if visual inspection of the accelerometer data from the Q-sensor indicated a significant amount of motion corresponded with significant motion artifacts in the EDA data during the timepoints of interest. Consistent with previous research from our team (8, 9), baseline EDA was determined by taking the median of 5 min of EDA data during the beginning of each session. Baseline periods did not include problem behaviors. Median baseline EDA was used instead of mean baseline EDA as the data were typically skewed in a negative direction which would influence mean scores. Next, the anticipatory EDA rise time was determined by examining EDA data 5 min prior to the onset of the problem behavior. An anticipatory rise in EDA was defined by the presence of at least two skin conductance responses of at least 0.03µS, followed by a gradual increase in the slope of EDA after the SCR during the 5-min period prior to the problem behavior. This method was chosen as it involves both tonic and phasic elements of skin conductance such that a skin conductance response that was due to motion was unlikely to satisfy the criteria for an anticipatory rise in EDA as it likely wouldn’t be followed by an increase in the slope of the EDA. A threshold of 0.03µS and even as low as 0.01μS has been shown to be common in the literature as a skin conductance response (23, 25). Next, latency to return to baseline EDA after the student engaged in problem behavior was determined by examining the amount of time between the point in time where the student stopped engaging in the problem behavior and the point where the EDA returned to the median baseline score. EDA records were also monitored for whether or not they returned to their median baseline EDA level, with an average duration of monitoring for return to baseline of at least 20 min (average duration = 134 min, ± 72 min. std. dev., range 20–242 min.) after engaging in problem behavior, and so frequency of this occurrence was tracked within each individual and for the overall group of students.

### Analysis

Frequencies of BIP and EDA associated with the behaviors were reported overall for each student. Incidence of anticipatory rise prior to behaviors and recovery of EDA after behaviors was also documented.

## Results

### Frequency of Problem Behaviors

**Figure 1:**
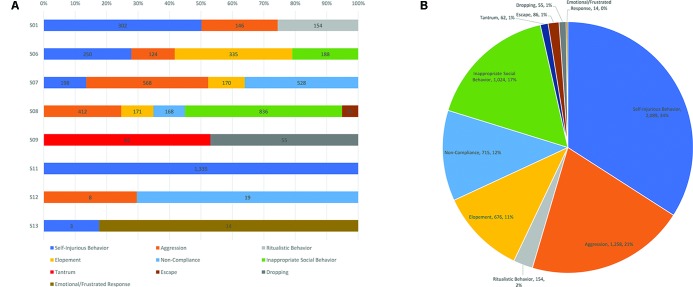
**(A)** Frequency counts of the types of problem behaviors engaged in over 1 year for each student in the sample. **(B)** Total number of behaviors engaged in by the sample over 1 year and proportion of the whole that each behavior represents.

To demonstrate the range of behaviors among the students, behavior frequency each participant engaged in over the year-long period assessed is depicted in **Figure 1**. Overall, most of the 8 students engaged in more than one type of behavior, except for student 11 who only engaged in self-injurious behavior. For all of the documented behaviors across all of the students, the most common problem behavior was self-injurious behavior, followed by aggression, inappropriate social behavior, non-compliance, and elopement.

### Quality of EDA Signal

Initially, the EDA data were visually inspected by an experienced psychophysiology researcher (BF) for artifacts as the Q-sensors were placed either on the wrist or the ankle of the students. Any cases with significant artifacts in EDA during the times that were analyzed, as defined by a visual analysis of motion from accelerometer data in the x, y, and z directions at the same time as significant EDA artifacts were noted, or DC shift, indicating either loose or no contact between the skin and the Q-sensor (25), were excluded from the final analysis. Significant EDA artifacts were defined as an immediate drop in EDA to 0, indicating DC shift, or when “spikes” or rapid increases in EDA data appeared and were at least three standard deviations above the mean. This typically corresponded with rapid fluctuations in accelerometer data in x, y, and z directions and was present during periods when the student engaged in problem behavior that resulted in movement of the arms and/or legs. Artifacts in the EDA data were easily detected as they tended to be characterized by quick “spikes” in the EDA data that aren’t physiologically likely, followed by DC shift, indicating that contact between the EDA sensor and the skin was broken. Visual inspection of each EDA record yielded 22 records that contained a significant amount of artifact, according to the visual analysis mentioned above, that rendered the data unreliable, and so they were excluded from the final analysis. Each of these 22 records were associated with participant behaviors. This yielded a total of 62 valid records that were suitable for analysis.

### Anticipatory Rise Time Prior to Problem Behavior

The presence or absence of an anticipatory rise in EDA prior to a student engaging in problem behavior was analyzed. On average, across all episodes of problem behavior documented, across all students, the students displayed an anticipatory rise in EDA prior to engaging in problem behavior 60% of the time. However, individuals varied in the frequency with which an anticipatory rise in EDA was observed before the onset of the problem behavior (see **Table 2**). Of note, the students with the greatest incidence of anticipatory rise in EDA had primarily engaged in general classroom disruption behaviors and self-injurious behavior, while the student with the least amount of times of an anticipatory rise had primarily engaged in aggression (see **Table 2**).

### Recovery of EDA to Baseline After Problem Behavior

On average, across all problem behaviors documented, the students’ EDA returned to median baseline values 45% of the time after engaging in problem behavior. Individuals also varied in the frequency with which EDA returned to median baseline levels after cessation of the problem behavior. Of note, the student who returned the most frequently to their median baseline EDA primarily engaged in out-of-seat behavior, while the student who returned the least amount of times to their median baseline EDA primarily displayed agitation (see **Table 2**).

**Figure 2:**
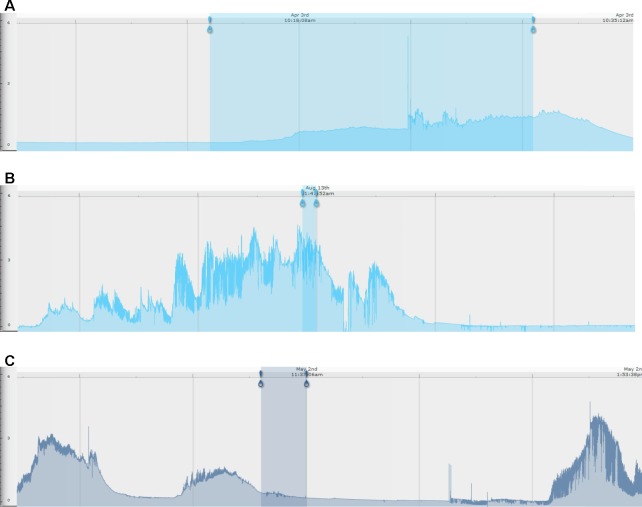
Examples of skin conductance level traces by behavioral subtype. For all images, the blue-shaded area represents the time period of an individual engaging in problem behavior. The left-most blue pin represents the beginning of the problem behavior, and the right-most pin represents the end of the problem behavior. **(A)** No EDA build-up prior to engaging in problem behavior (in this example, jumping in seat); **(B)** EDA build-up prior to engaging in problem behavior (in this example, jumping in seat); **(C)** EDA build-up prior to engaging in problem behavior (in this example, repetitive body hitting) and subsequent reduction with engagement in problem behavior. The x-axis is EDA in microsiemens, and the y-axis is time (marked at the top of each EDA record).

Examples of the different types of EDA responses are illustrated in **Figure 2**. For some problem behavior, the EDA pattern was characterized by a gradual increase in EDA leading up to the problem behavior, a peak in EDA while the individual was engaged in the problem behavior, and then a gradual decrease in EDA following discontinuation of the problem behavior. For some problem behavior, the EDA pattern was characterized by no build-up in EDA prior to the engagement of the problem behavior, with a gradual increase in EDA during engagement in the problem behavior, followed by a decrease in EDA following discontinuation in the behavior. For other episodes of problem behavior, the EDA pattern was characterized by an increase in EDA prior to engagement in the problem behavior which was followed by a decrease in EDA during engagement of the problem behavior (see **Figure 2** for examples of each response pattern).

## Discussion

The findings from this exploratory study suggest that examining the relationship between EDA and problem behaviors is feasible in a naturalistic setting in a severely affected population with ASD. There was some loss of data due to artifact, which was expected due to the level of functioning of the students and the assessment of EDA prior to and during the engagement of problem behavior that involves motion, rather than in a well-controlled laboratory setting. However, given the exploratory nature of the study, the findings suggest that the collection of EDA in a population with severe ASD is possible, but investigators should be aware of a number of limitations that are likely to occur in this population.

Variability was found in the incidence of changes in EDA prior to the occurrence of problem behavior, during the problem behavior, and after the occurrence of a problem behavior, targeting a unique set of severely affected individuals in a residential setting. The results indicate that 60% of the total episodes of problem behavior documented were associated with an anticipatory rise in EDA prior to engaging in problem behavior. This has important implications for efforts to utilize psychophysiological markers as a predictor of problem behaviors (14). This finding could also have significant implications for the prediction and management of problem behavior in a classroom setting of individuals who are severely affected by ASD. For those cases where an anticipatory rise was found, the average amount of time that EDA rose prior to engaging in problem behavior was over 10 min, providing a window of opportunity for intervention to occur prior to the occurrence of problem behavior. However, this would not be helpful in all cases, as many behaviors were not associated with an anticipatory rise in EDA. Further monitoring of these individuals may be helpful to understand other factors that might predict behaviors, and in how many of these individuals do other psychophysiological variables contribute predictive information. Given the variability in behavioral profiles of individuals with ASD in this study, future work will need to determine whether specific behavioral profiles are associated with certain patterns of EDA change, allowing improved specificity for the psychophysical prediction efforts. Many have suggested that understanding the psychophysiological underpinnings of problem behavior in ASD is important for predicting when an individual with ASD is likely to engage in problem behaviour (12, 26), but the distinct patterns of EDA suggest that different treatment strategies may be more effective for each type of EDA response pattern. For example, individuals with ASD that display a steady increase in EDA prior to engaging in problem behavior may respond best from cognitive and/or behavioral interventions to reduce anxiety. Additionally, pharmacological treatment with agents targeting stress reactivity may also provide benefit in this subtype. For example, propranolol, an agent utilized in other conditions associated with altered stress reactivity (27), has been shown to increase conversational reciprocity in ASD (9), making it a potential candidate drug for the treatment of anxiety-related behaviors in ASD. However, those without an increase in EDA prior to engaging in problem behavior may benefit from behavioral or pharmacological interventions targeting impulse control.

In this sample, we also identified that over half of the students did not reliably return to their initial resting baseline EDA after engaging in problem behavior. This suggests that, in future work examining the relationship between EDA and behaviors, it will be critical to account for the potential confound of a lack of returning to baseline from previous behaviors for EDA, and which behavioral profiles are associated with lack of return to baseline. Additionally, this raises the possibility that interventions after the cessation of behavior might be beneficial in some individuals, targeting de-escalation.

A number of limitations to the present study should be noted, as they affect the generalization of the results across a broad range of individuals with ASD. First, the students in this study ranged in age from 13 to 20 years, with an average age of 16, were all male and have severe ASD. As such, it is not clear how this generalizes to all individuals with ASD, and so future studies will need to examine data from a more diverse sample of individuals. Second, detailed data from only eight individuals were analyzed for this study, and so future research should aim to examine these results in a much larger group of individuals with ASD, which would also allow the examination of the impact of co-occurring conditions and medications on the EDA/problem behavior relationship. However, this remains of interest for the management of those with the most severe problem behaviors, who were studied herein, and the variability that will be important in future psychophysiological monitoring in this type of setting.

Finally, when planning psychophysiological experiments in those with severe ASD, careful consideration should be given to the testing environment, the problem behaviors engaged in by the individual(s) to be studied, and the tolerance of biosensors on the individual. For instance, if data collection will be indoors and outdoors, EDA would not be an appropriate measure given that EDA readings can be influenced by changes in hydration status, relative humidity, or sweat, for example (23, 28, 29). In this case, it may be better to collect electrocardiogram (ECG) to analyze heart rate variability (HRV), which provides information about sympathetic as well as parasympathetic nervous system functioning. Further, if an individual engages in arm flapping or elopement—for example, collection of data from the wrist or leg may not be appropriate given the high probability that the data will contain motion artifact. To this point, EDA data from student 13 in this study was unable to be analyzed due to significant motion artifact from repetitive motor movements (**Table 2**). In this case, investigators may consider the use of a physiological apparatus that is affixed to the trunk of the body that is less susceptible to motion artifact. As such, there are a number of limitations of collecting EDA data from those with severe ASD, but with careful planning, such studies are possible and add a wealth of knowledge on those more severely affected.

## Data Availability

The datasets generated for this study are available on request to the corresponding author.

## Ethics Statement

This study was carried out in accordance with the recommendations of the Institutional Review Board (IRB) at The Center for Discovery with written informed consent from all subjects. All subjects gave written informed consent in accordance with the Declaration of Helsinki. The protocol was approved by the IRB at The Center for Discovery. Data analyses were conducted at the University of Missouri under a reliance agreement between the University of Missouri Health Sciences Institutional Review Board and IRB at The Center for Discovery.

## Author Contributions

BF conceptualized and designed the study, coordinated and supervised data management, analyzed the EDA data, drafted the initial manuscript, and reviewed and revised the manuscript. JL and TV collected and processed the initial data, and assisted with preparation of the manuscript. JC advised on the data handling and assisted with preparation of the manuscript. TH developed the program for data collection, supervised data collection at The Center for Discovery, and revised the manuscript. DB conceptualized the overall plan of the study with BF, supervised the research team and provided expertise and guidance regarding autism spectrum disorder and revised the manuscript. All authors approved of the final manuscript as submitted.

## Funding

This study was funded by the New York State Center of Excellence grant funds from the New York State Department of Health & Office for People with Developmental Disabilities (OPWDD), & private gifts provided to The Center for Discovery.

## Conflict of Interest Statement

DB is on the research advisory board for The Center for Discovery, where TH, JL, JC, and TV are employed. The remaining authors declare that the research was conducted in the absence of any commercial or financial relationships that could be construed as a potential conflict of interest.

## Abbreviations

ASD, autism spectrum disorder; EDA, electrodermal activity; TCFD, The Center for Discovery; ASRS, Autism Spectrum Rating Scales; DSM-IV-TR, Diagnostic and Statistical Manual of Mental Disorders IV—Text Revision; IQ, intelligence quotient; ABAS, Adaptive Behavior Assessment System; VABS, Vineland Adaptive Behavior Scales; CTONI-2, Comprehensive Test of Nonverbal Intelligence—Second Edition; std. dev, standard deviation.
